# Leader cell PLCγ1 activation during keratinocyte collective migration is induced by EGFR localization and clustering

**DOI:** 10.1002/btm2.10138

**Published:** 2019-06-26

**Authors:** Chloe S. Kim, Xinhai Yang, Sarah Jacobsen, Kristyn S. Masters, Pamela K. Kreeger

**Affiliations:** ^1^ Department of Biomedical Engineering University of Wisconsin—Madison Madison WI 53705; ^2^ Carbone Cancer Center University of Wisconsin School of Medicine and Public Health Madison WI 53705; ^3^ Department of Medicine University of Wisconsin School of Medicine and Public Health Madison WI 53705; ^4^ Department of Cell and Regenerative Biology University of Wisconsin School of Medicine and Public Health Madison WI 53705

**Keywords:** immobilized growth factor, receptor clustering, re‐epithelialization, wound healing

## Abstract

Re‐epithelialization is a critical step in wound healing and results from the collective migration of keratinocytes. Previous work demonstrated that immobilized, but not soluble, epidermal growth factor (EGF) resulted in leader cell‐specific activation of phospholipase C gamma 1 (PLCγ1) in HaCaT keratinocytes, and that this PLCγ1 activation was necessary to drive persistent cell migration. To determine the mechanism responsible for wound edge‐localized PLCγ1 activation, we examined differences in cell area, cell–cell interactions, and EGF receptor (EGFR) localization between wound edge and bulk cells treated with vehicle, soluble EGF, or immobilized EGF. Our results support a multistep mechanism where EGFR translocation from the lateral membrane to the basolateral/basal membrane allows clustering in response to immobilized EGF. This analysis of factors regulating PLCγ1 activation is a crucial step toward developing therapies or wound dressings capable of modulating this signal and, consequently, cell migration.

**Abbreviations**
ECM: extracellular matrix
EGF: epidermal growth factor
FBS: fetal bovine serum
PBS: phosphate buffered saline
PLCγ1: phospholipase C gamma 1
TGFβ: transforming growth factor β

## 1 INTRODUCTION

Collective migration results from the coordinated movement of cells that are in physical contact. This form of migration is crucial for embryonic development, metastasis, and dermal wound healing, where it is responsible for re‐epithelialization.^1^ When a wound occurs in a confluent sheet of cells, the cells experience an intrinsic polarity based on the localization of cell–cell contacts at the trailing edge, which allows for cell protrusions to be extended into the open space to initiate migration.^2^ These “leader cells” then pull the cells from the bulk along through coordinated cell–cell connections. Impairment of this process leads to the development of chronic wounds, such as those associated with diabetes.^3^

Cells at different locations within the collective sheet display different phenotypes during collective migration.^4^, ^5^ Once established, leader cells are crucial for coordinated migration and exhibit different signaling patterns, such as elevated Rac1 and PI3K activity.^5^ Variations in the microenvironment such as extracellular matrix (ECM) density or the presence of gradients of soluble growth factors can induce or reinforce the behaviors of leader cells.^2^, ^6^ However, our work and others have demonstrated that keratinocytes also exhibit increased collective migration and differential signaling in leader cells when treated with a uniform concentration of growth factors in vitro. For example, epidermal growth factor (EGF) and transforming growth factor β1 (TGFβ1) are both found in the wound microenvironment and induce re‐epithelialization in vitro.^7^ Our findings revealed that EGF‐induced wound closure was dependent on the manner of growth factor presentation, with wounds closing significantly faster on EGF covalently immobilized to the culture substrate, a presentation that mimics ECM‐entrapped growth factors.^8^ The increased closure obtained with immobilized EGF was a result of individual cells at the leading edge having increased migrational persistence and directionality into the wound.^9^ We determined that this unique migratory phenotype resulted from the activation of phospholipase C gamma 1 (PLCγ1), which was highly specific to the leader cells and observed only in response to immobilized EGF.^9^ Similarly, Chapnick and Liu recently demonstrated that spatial restriction of ERK activation to the leading edge of keratinocytes resulted in highly directional migration reminiscent of TGFβ stimulation.^10^ However, while both of these studies highlight that unique intracellular signaling in leader cells has phenotypic consequences for wound healing, neither study determined what conditions or properties enabled this localized signaling. Here, we examine several hypotheses and identify differences in receptor localization and clustering as responsible for the spatial restriction of pPLCγ1 to the leader cells on immobilized EGF. Elucidation of these mechanisms provides insight into wound biology, as well as important guidance for the development of biomaterials‐ or tissue engineering‐based strategies to improve re‐epithelialization.

## 2 RESULTS AND DISCUSSION

### 2.1 Immobilized EGF increases extent of PLCγ1 activation

**Figure 1:**
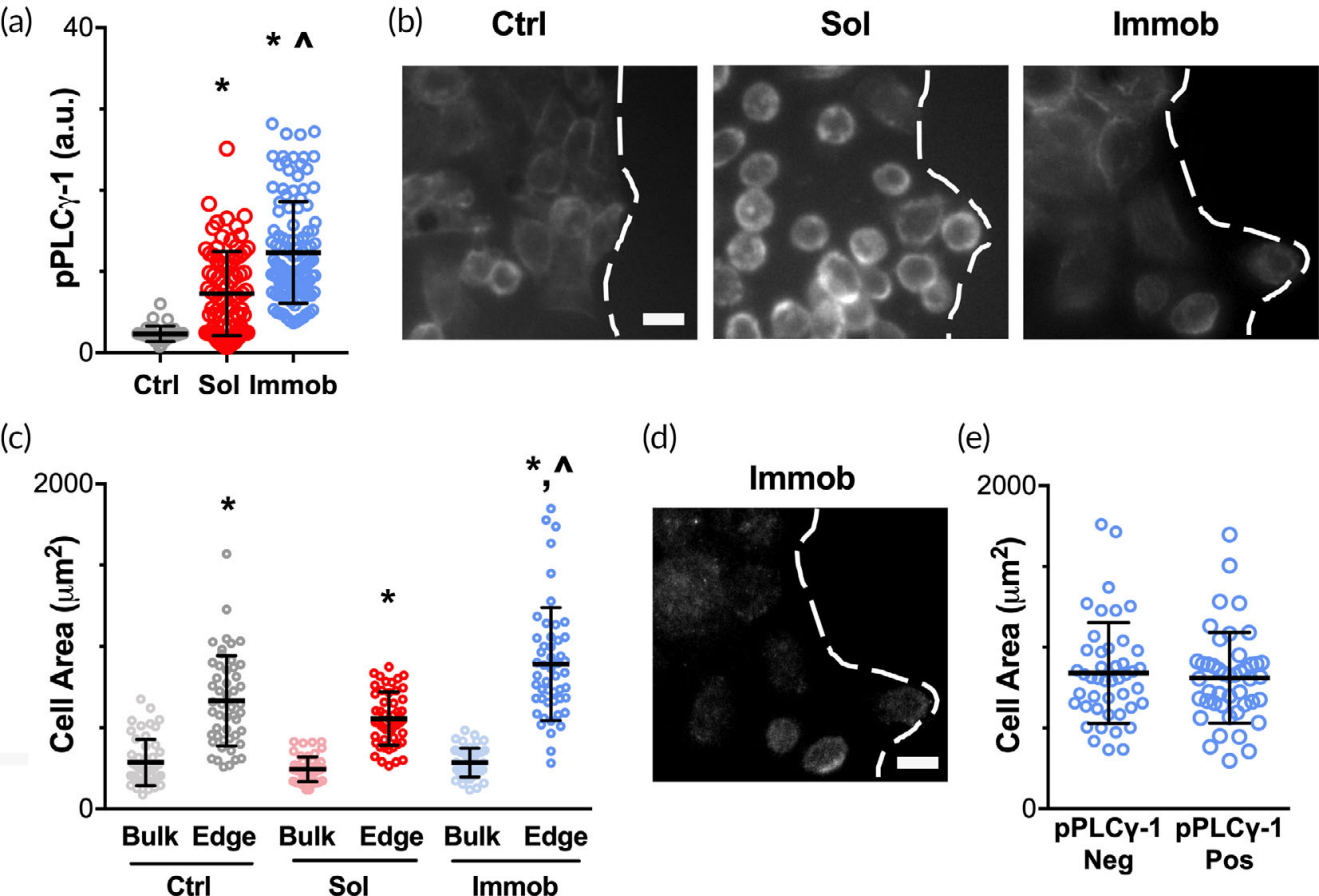
pPLCγ1 activation is not due to variations in cell area. (a) HaCaTs were seeded as confluent monolayers and treated with vehicle control, soluble EGF, or immobilized EGF for 4 hr after the fence was lifted, and then stained for pPLCγ1 (representative images provided in Supporting Information Figure 1). pPLCγ1 intensity was quantified in cells adjacent to the wound edge. Data presented as individual cells (*n* = 35–116 cells/condition) with mean ± *SD* shown as lines. * indicates significantly different relative to control; ^∧^ indicates significantly different relative to soluble by Tukey‐HSD, *p* < .05. (b) HaCaTs were stained with phalloidin to visualize actin filaments after 4 hr of treatment. Dashed lines indicate wound edge, scale bar = 50 μm. (c) Cell area was quantified based on the cortical actin outline for cells in the bulk and on the edge. Data presented as individual cells (*n* = 50 cells/condition), with mean ± *SD* shown as lines. * indicates significantly different relative to bulk for same condition; ^∧^ indicates significantly different relative to control and soluble edge by Tukey‐HSD, *p* < .05. (d) pPLCγ1 staining for immobilized EGF condition shown in (b). Dashed line indicates wound edge, scale bar = 50 μm. (e) Cell area was not significantly different for pPLCγ1 positive and negative cells on immobilized EGF. Data presented as individual cells (*n* = 45 cells/group), with mean ± *SD* shown as lines

We have previously demonstrated that the number of pPLCγ1 positive cells is significantly increased for cells on immobilized EGF along the wound edge relative to both cells in the bulk and to cells exposed to control conditions or an equivalent dose of soluble EGF.^9^ To further probe this finding, we analyzed the average pPLCγ1 intensity for cells along the wound edge for HaCaTs treated with soluble EGF or immobilized EGF at 4 hr. As expected, the pPLCγ1 intensity was comparable to background levels in control conditions, while pPLCγ1 intensity was significantly elevated by treatment with EGF and was further elevated when cells were exposed to immobilized rather than soluble EGF (Figure 1a, Supporting Information Figure 1). Therefore, in addition to our prior finding that more edge cells demonstrate clear activation of PLCγ1 on immobilized EGF compared to soluble EGF,^9^ the extent of activation within an individual cell is also increased with immobilized EGF.

### 2.2 PLCγ1 activation does not result from larger cell areas observed near the wound edge

To understand why the cells on the edge have increased activation of pPLCγ1, we considered some of the prior explanations for wound edge‐specific behavior as well as differences between the edge and bulk cells. Edge‐specific activation of leader cells has been observed in response to chemotactic gradients^6^; however, all of the cells in the keratinocyte sheet in our experiments were exposed to a uniform concentration of immobilized EGF. It is widely recognized that keratinocytes at the wound edge undergo hypertrophy,^11^, ^12^ and previous reports using uniform stimuli have linked differences in leader cell signaling to variations in cell size^13^ or the related property of cell density.^10^ To examine the possibility that cell size regulates the edge‐specific PLCγ1 phosphorylation found in HaCaTs treated with immobilized EGF (Figure 1b), cell areas were measured based on actin staining. Consistent with prior studies,^11^ cells on the wound edge had larger areas (Figure 1c). In addition, cells on immobilized EGF had significantly larger cell area at the edge when compared to all other groups, suggesting a possible link between cell area and PLCγ1 activation. However, the distribution of cell sizes between all conditions overlapped; in particular, edge cells on immobilized EGF were only slightly larger than edge cells treated with soluble EGF. Therefore, we conducted a detailed analysis of the edge cells on immobilized EGF (Figure 1d) and determined that there was not a significant difference in cell area between pPLCγ1‐positive and pPLCγ1‐negative cells (Figure 1e). This result suggests that increased cell area was not responsible for the increased activation of pPLCγ1 on immobilized EGF.

### 2.3 PLCγ1 activation requires a decrease in tight junctions with neighboring cells

**Figure 2:**
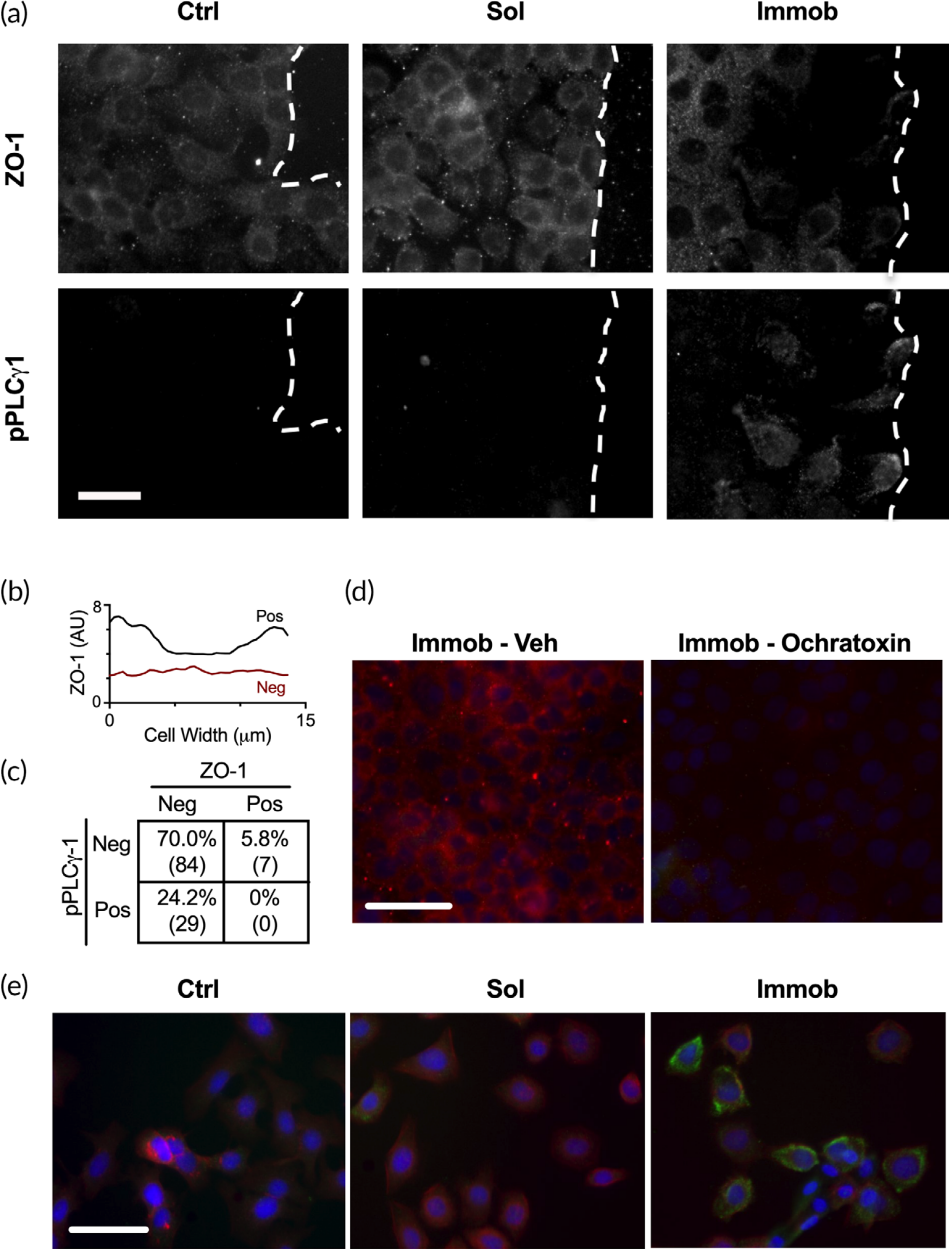
Loss of ZO‐1 is necessary, but not sufficient, for PLCγ1 activation. (a) Cells from all three treatment conditions in the wound model were examined for ZO‐1 (top) and pPLCγ1 (bottom) after 4 hr of treatment. Scale bar = 20 μm; dashed line indicates wound edge. (b) The fluorescent intensity was measured across the cell diameter to determine the ratio between the cell membrane and cytoplasm; ZO‐1 positive cells had a ratio greater than or equal to 1.5 and ZO‐1 negative cells had a ratio less than 1.5. (c) Quantification of pPLCγ1 and ZO‐1 status for *n* = 120 cells on immobilized EGF, significantly different by chi‐square analysis, *p* < .05. (d) Cells on immobilized EGF were treated with vehicle or ochratoxin and stained for ZO‐1 (red), pPLCγ1 (green), and nuclei (blue). Loss of ZO‐1 from cells in the bulk was insufficient to induce PLCγ1 activation. (e) Cells were seeded at a low density to limit the number of neighboring cell contacts and stained as in (d) after 4 hr of treatment. Scale bar = 50 μm in (d) and (e)

We next examined whether differences in cell–cell connections could play a role in the observed activation of PLCγ1, as cells at the leading edge need to remodel their tight junctions in order to migrate.^14^ Epidermal growth factor receptor (EGFR) activation has been shown to increase tight junction assembly in confluent cells,^15^ but did not impact zonula occludens‐1 (ZO‐1) expression or localization.^16^ Alternatively, cytokines that disrupt tight junctions in airway epithelial cells do so through EGFR activation of ERK.^17^ However, the role of tight junctions in PLCγ1 activation is not known. Cells were co‐stained for ZO‐1, one component of tight junctions in keratinocytes,^18^ and pPLCγ1 (Figure 2a). Cells were quantified as pPLCγ1‐positive and as ZO‐1 positive based on the ratio of membrane: cytoplasmic signal (Figure 2b). This classification demonstrated that all cells that were pPLCγ1‐positive were also ZO‐1 negative, and that this was significantly different compared to a random distribution (Figure 2c). To determine if loss of tight junctions was sufficient to induce pPLCγ1 in cells located in the bulk, cells on immobilized EGF were treated with ochratoxin‐A, a mycotoxin that has previously been shown to disrupt tight junctions.^19^ As expected, treatment with ochratoxin‐A resulted in a shift in ZO‐1 staining from membrane‐localized to diffuse or nearly absent throughout the cell body for bulk cells (Figure 2d). However, there was no increase in pPLCγ1 staining in the bulk despite the loss of tight junctions, suggesting that breakdown of tight junctions alone was not sufficient to induce activation. This may indicate that other cell–cell junctions that are unaffected by ochratoxin are involved in the mechanism. Therefore, we plated cells at a low seeding density and stimulated them with EGF before they had time to reach confluency and form strong cell–cell junctions (Figure 2e). In this setting, pPLCγ1 was again only observed when cells were cultured on immobilized EGF, but not all cells in this condition were pPLCγ1‐positive. Therefore, we conclude that the loss of ZO‐1 containing tight junctions is necessary, but not sufficient, for PLCγ1 activation.

### 2.4 EGFR localization varies between edge and bulk cells

**Figure 3:**
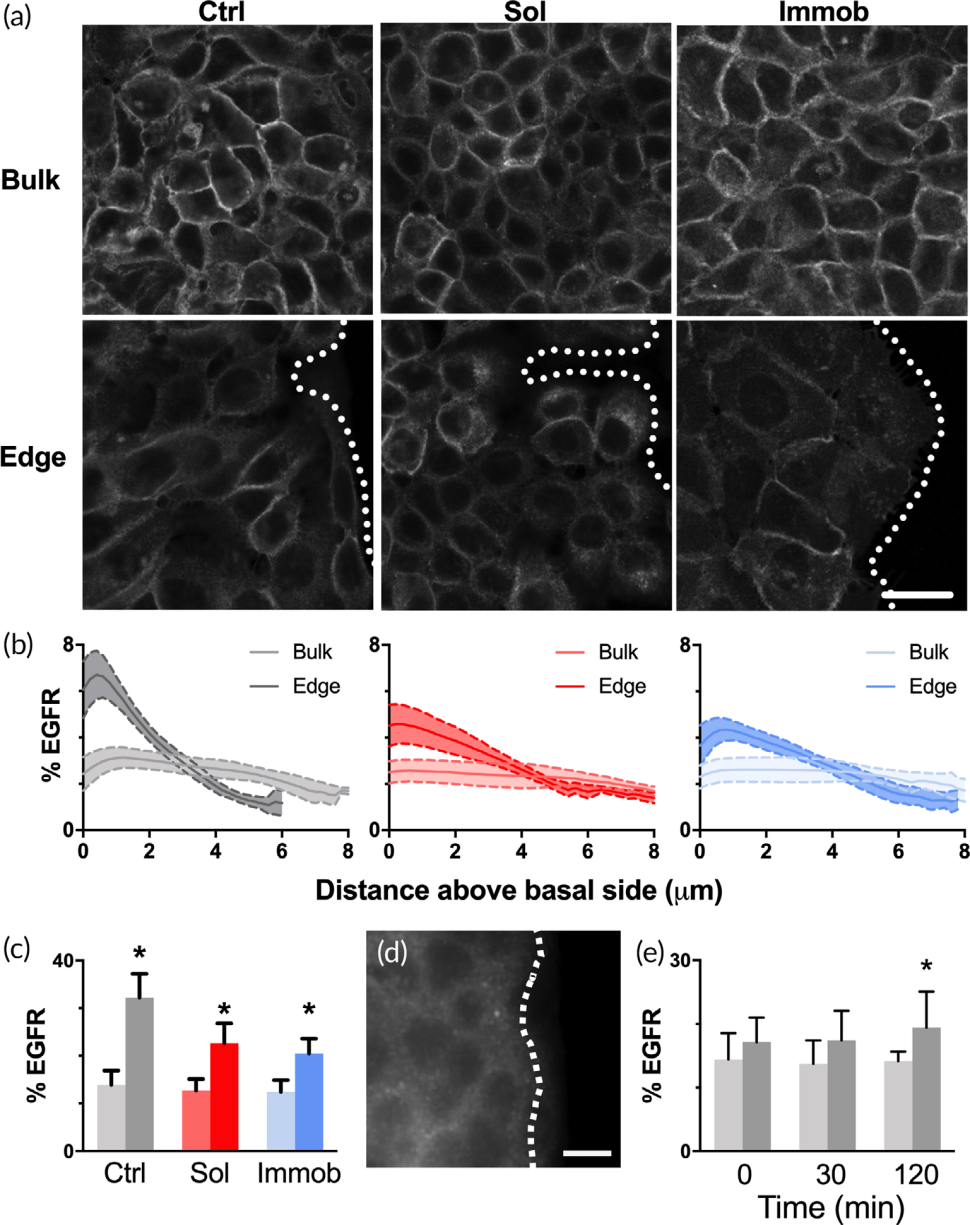
EGFR is localized to the basal membrane for cells along the wound edge. (a) Cells from all three treatment conditions in the wound model were examined at 4 hr for EGFR by confocal microscopy. Shown are the z‐sections from the basal membrane/substrate interface. Scale bar = 25 μm; dashed line indicates wound edge. (b) Z‐stack reconstructions for individual cells were analyzed to determine the percentage of the total EGFR signal located at each plane from the basal (0 μm) to the apical side at the wound edge and in the bulk. Dark lines in the middle represent mean and light shadings show *SD*, *n* = 15–20 cells/condition. Note that cells in different conditions had different heights. (c) Percentage of EGFR within 1 μm of the basal membrane for conditions in (b). Data represented as mean ± *SD*, *n* = 15 cells/condition. * indicates significantly different relative to bulk of same condition by Sidak's multiple comparison, *p* < .05. (d) ZO‐1 staining at the time of fence removal demonstrated that tight junctions on the wound edge were intact initially. Dashed line indicates wound edge, scale bar = 10 μm. (e) Percentage of EGFR within 1 μm of the basal membrane at the edge and bulk in control conditions from time of fence removal to 2 hr later. Data represented as mean ± *SD*, *n* = 15–20 cells/condition, * indicates *p* < .05 relative to bulk at same time by Sidak's multiple comparison test

Tight junctions serve as adhesive contacts among epithelial cells and restrict membrane protein movement between apical and basolateral membranes.^20^ Therefore, we next examined whether EGFR localization varied between cells along the leading edge and cells in the bulk. Cells in all three conditions (control, soluble EGF, immobilized EGF) were examined by confocal microscopy for EGFR (Figure 3a), and z‐stack reconstructions analyzed for patterns in EGFR signal per cell. Other reports have suggested that leader cells are associated with differences in the levels of cell surface receptors, which could lead to increased activation in response to a stimulus.^5^ Examination of the images suggested that EGFR may be lower at the wound edge; however, due to differences in cell size (i.e., larger area at the edge) it was possible that cells with a weaker intensity/pixel had the same or more EGFR when integrated across the cell volume. In our system, the integrated intensity for EGFR over the entire z‐stack of the cell was less than 5% different for edge versus bulk cells (data not shown), suggesting there was not a major difference in expression level. However, when the percentage of the total EGFR signal per cell located in each plane (from the basal to the apical side) was quantified, a dramatic difference in protein localization was observed (Figure 3b). For all three treatments, cells in the bulk had evenly dispersed EGFR throughout the height of the cell. In contrast, cells located on the wound edge showed a significantly increased level of EGFR in the planes closest to the basal membrane (Figure 3c); this pattern did not vary with EGF treatment (*p* = .31, soluble edge vs. immobilized edge). When examined at the time of fence removal, ZO‐1 was observed to be intact in edge cells (Figure 3d) and EGFR basal localization was not different between edge and bulk cells (Figure 3e). After 2 hr, the level of basal EGFR was significantly increased relative to bulk cells, suggesting that EGFR relocation occurs either concurrent with or following the release of cellular junctions. This mechanism of differential receptor localization provides another potential mechanism to regulate leader cell‐specific behaviors. Indeed, localization of EGFR to the basal side may play an important role in native wound healing, as the sources of EGF in the wound microenvironment (e.g., macrophages^21^) are located under the keratinocyte sheet. The basal localization of EGFR may also limit the responsiveness of keratinocytes to topically‐applied EGF in wound treatment, potentially explaining the poor efficacy of this approach.^22^

### 2.5 EGFR clustering is induced on immobilized EGF

While the differences in ZO‐1 and EGFR localization explain why cells in the bulk did not have active PLCγ1, it does not explain the specificity for immobilized versus soluble EGF. Understanding why cells do not respond to soluble EGF may provide insight into co‐treatment strategies to improve the efficacy of soluble growth factors, which are easier to deliver. While the pattern of EGFR vertical localization was consistent across all three treatments, the distribution of EGFR along the basal membrane varied with both cell location and treatment. As shown in the images from the basal plane (Figure 3a), cells in the bulk tended to have EGFR signal localized to the lateral membrane boundary, while cells along the edge had more EGFR diffused throughout the plane where the cell contacts the culture surface. Notably, EGFR signal was more punctate on immobilized EGF, suggesting that receptor clustering may be unique to this condition.

**Figure 4:**
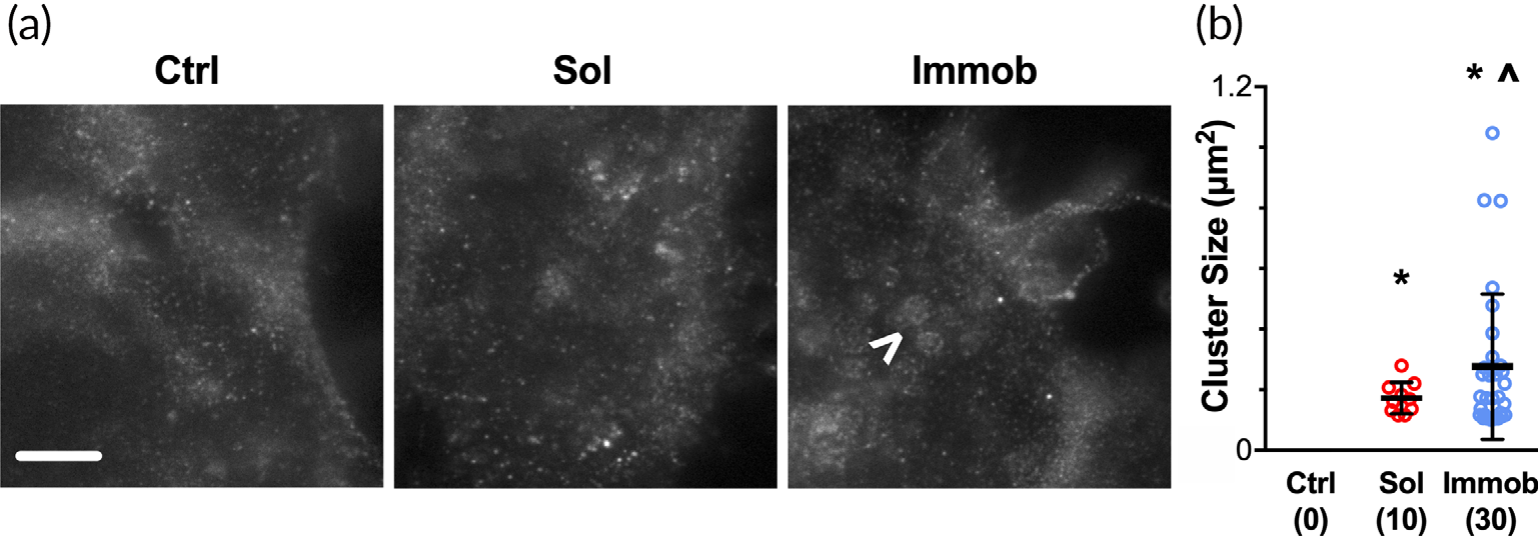
STED imaging reveals larger EGFR clusters on edge cells on immobilized EGF. (a) Cells from all three treatment conditions in the wound model were examined by STED for EGFR at 4 hr. Arrowhead points to a large EGFR cluster observed with immobilized EGF. Scale bar = 3 μm. (b) Quantification of EGFR cluster sizes for each condition. Data presented as individual clusters with the number of clusters noted for each condition and mean ± *SD* shown as lines, * indicates significantly different relative to threshold of 0.1 μm^2^ by one‐sample *t* test, *p* < .05; ^∧^ indicates significantly different relative to soluble by *t* test with Welch's correction for unequal variance, *p* < .05

Based on the observed differences in EGFR staining using confocal imaging, we next imaged the basal membrane for EGFR using stimulated emission depletion microscopy (STED) in order to gain improved resolution and characterize patterns in cluster size. In the bulk, EGFR was localized primarily to the basolateral junction, and the signal was evenly distributed with few apparent regions of higher concentration (Supporting Information Figure 2). For cells on the wound edge, the distribution of EGFR in control conditions was not restricted to the basolateral membrane, but appeared as scattered EGFR signal without obvious clusters (Figure 4a). Cells treated with soluble EGF showed some areas of potential receptor clusters, while cells on immobilized EGF had large areas of dense EGFR signal (Figure 4a). Previous studies have defined receptor clusters as ranging from 0.07 to 3 μm^2^ 23, 24; therefore, we classified any signal with an area less than 0.1 μm^2^ as an isolated receptor and quantified the sizes of all clusters (Figure 4b). Few EGFR clusters were observed in cells in the bulk regardless of treatment condition (Supporting Information Figure 2); in addition, cells in the control condition had no observable receptor clusters at the wound edge. Cells on the wound edge that were treated with soluble EGF had EGFR clusters with a size of approximately 0.1–0.3 μm^2^, which was significantly greater than the minimum threshold level. Edge cells on immobilized EGF contained EGFR clusters that were significantly larger than the minimum threshold, ranging from 0.1 to 1.0 μm^2^, and were also significantly larger than clusters observed with soluble EGF. Combined, the difference in cluster pattern between edge cells on immobilized EGF and all other conditions suggests that the increased basal receptor concentration observed in edge cells (Figure 3), in combination with growth factor presentation in a tethered form, enables receptor clustering. Clustering of the EGFR at the plasma membrane has been observed in tumor cells^25^ and in response to EGF treatment,^24^ and results in EGF signal amplification and duration. Although the linker length used in the current work (0.18 nm) is shorter than tethers used in previous studies aimed at inducing receptor clustering,^26^ the dense distribution of immobilized EGF (10 nm spacing between molecules) and the high expression of EGFR by HaCaTs (approximately 10^6^ receptors/cell^8^, ^27^) in our system were likely sufficient to enable receptor clustering when the receptors were concentrated at the basal surface for cells along the wound edge.

### 2.6 Cholesterol depletion promotes EGFR oligomerization and PLCγ1 activation by EGF

**Figure 5:**
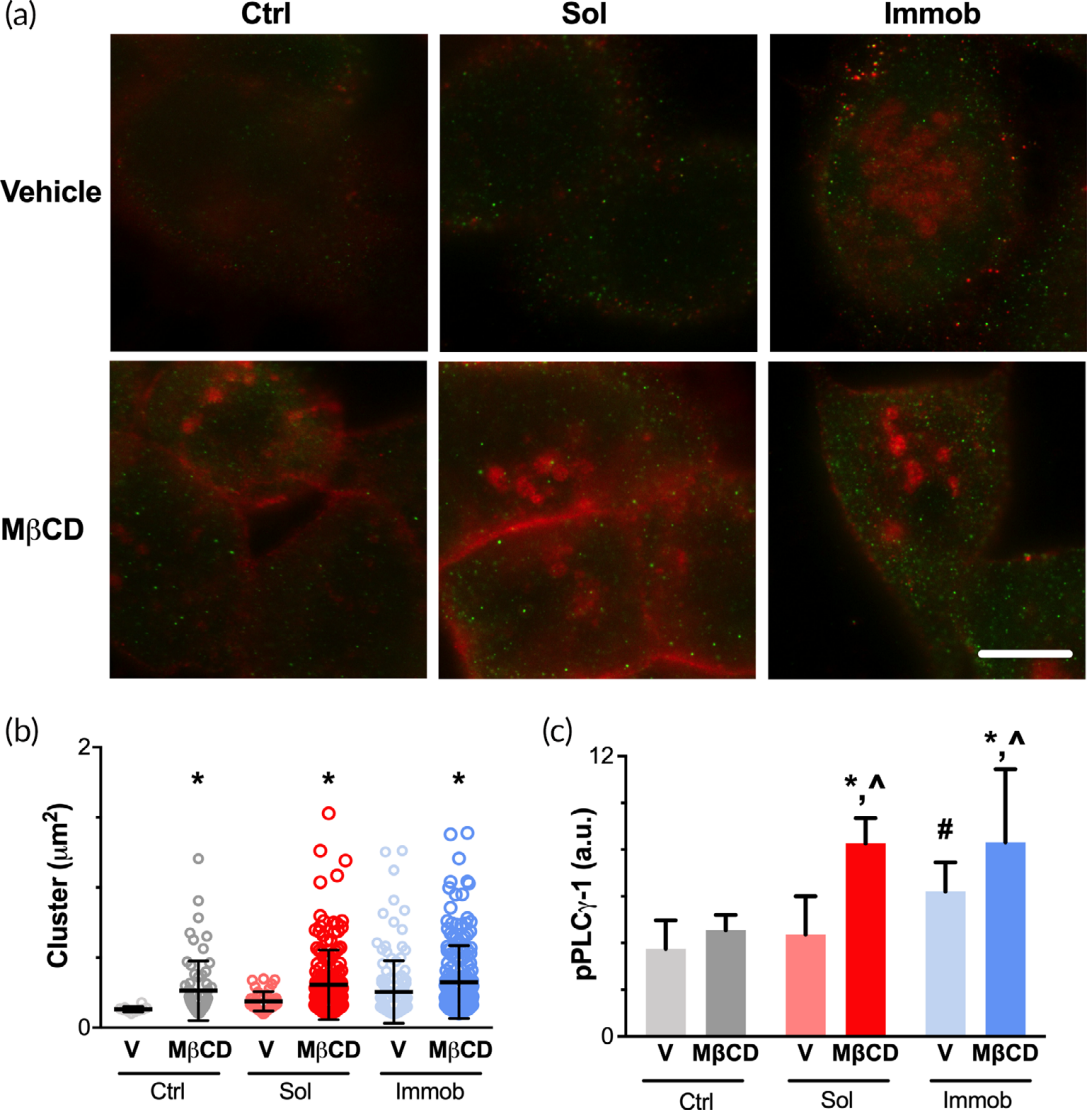
Methyl‐β‐cyclodextrin induced EGFR clustering and PLCγ1 activation in response to soluble EGF. (a) Cells were seeded at a low density and treated with vehicle (V) or 50 mM methyl‐β‐cyclodextrin (MβCD) for 4 hr. Cells were examined by STED for EGFR (red) and pPLCγ1 (green). Scale bar = 5 μm. (b) Quantification of EGFR cluster sizes for each condition. Data presented as individual clusters with the number of clusters noted for each condition and mean ± *SD* shown as lines. * indicates significantly different relative to vehicle control for that condition, *p* < .05 by *t* test with Welch's correction for unequal variance and Bonferroni correction for multiple comparison. (c) Quantification of pPLCγ‐1 fluorescent intensity in each condition. Data presented as mean ± *SD*, *n* = 9 images/condition. * indicates significantly different relative to vehicle with same EGF treatment; ^∧^ indicates significantly different relative to control with MβCD; # indicates significantly different relative to control with vehicle; *p* < .05 by Tukey‐HSD

As noted above, EGFR clusters were substantially larger in cells treated with immobilized EGF relative to soluble EGF, and, similar to the localization pattern observed for pPLCγ1, this clustering was localized to cells on the edge. Based upon these findings, we hypothesized that induction of EGFR clustering would enable soluble EGF to activate PLCγ1. Methyl‐β‐cyclodextrin is a cholesterol‐depleting agent that disrupts the lipid rafts on the plasma membrane^28^ and has previously been shown to induce EGFR clusters in HaCaTs.^29^ To maximize the number of cells that were capable of inducing PLCγ1, we examined HaCaTs plated at low density where tight junctions have not formed (Figure 2e). Cells were treated with vehicle or methyl‐β‐cyclodextrin in combination with control or soluble EGF, and then examined by STED to determine cluster size (Figure 5a, Supporting Information Figure 3). As expected, treatment with methyl‐β‐cyclodextrin resulted in the formation of both more clusters and significantly larger clusters in all conditions (Figure 5b). We next examined PLCγ1 activation and saw no difference in pPLCγ1 in the control condition, indicating that receptor clustering alone was insufficient to induce EGFR activation of PLCγ1 in the absence of ligand (Figure 5c). As predicted, pPLCγ1 was significantly increased in cultures treated with methyl‐β‐cyclodextrin in combination with soluble EGF. Likewise, methyl‐β‐cyclodextrin increased receptor oligomerization and pPLCγ1 levels for the immobilized EGF condition. These findings support our hypothesis that induction of EGFR clustering—either by ligand immobilization or through cholesterol depletion—enables EGF to activate PLCγ1 in HaCaTs.

### 2.7 Conclusions

Combined, our data suggest that differences in EGFR apical/basal localization coincident with the release of cellular junctions are responsible for the edge‐specific activation of pPLCγ1, while the ability to form EGFR clusters is responsible for the specificity for immobilized over soluble EGF. While we are unaware of previous work connecting EGFR clusters specifically to PLCγ1 activation, it is known that EGF family ligands have varying affinities for EGFR, with only some ligands having the ability to activate PLCγ1.^30^ Ligand affinity has also been shown to impact the stability of receptor dimers, with downstream effects on signal duration and cell fate.^31^ These prior findings are consistent with our finding of activation of PLCγ1 in response to EGFR clustering through either immobilized EGF or cholesterol depletion. Our previous work has demonstrated that migration persistence of individual keratinocytes within a collective cell sheet is the strongest predictor of in vitro wound closure^11^ and that PLCγ1 activation is responsible for this persistent movement.^9^ Thus, PLCγ1 may serve as a target for improving wound re‐epithelialization, and understanding the factors that lead to its activation is a crucial step toward developing therapies or wound dressings capable of modulating this signal. Additionally, PLCγ1 is recognized to impact motility in a wide range of cell types,^32^, ^33^, ^34^ therefore, elucidation of the factors that cause edge‐specific activation of PLCγ1 may translate to other cell types and tissues.

## 3 MATERIALS AND METHODS

All materials were purchased from Thermo Fisher Scientific (Waltham, MA) unless noted otherwise.

### 3.1 Cell culture

Immortalized human keratinocytes (HaCaT cells, courtesy of N. Fusenig, DKFZ, Heidelberg, Germany) were maintained at 37°C, 5% CO_2_ in high‐glucose Dulbecco's modified Eagle medium (DMEM) supplemented with 10% fetal bovine serum (FBS) and 1% penicillin/streptomycin. Cells were routinely screened for mycoplasma using MycoAlert (Lonza, Basel, Switzerland). To seed cells for experiments, HaCaTs were incubated with 0.05% ethylenediaminetetraacetic acid for 20 minutes at 37°C and 0.05% trypsin for 5 minutes at 37°C. Cells were neutralized with 0.5 mg/mL soybean trypsin inhibitor and resuspended in DMEM with 0.5% FBS, 1% penicillin/streptomycin and 2 μM AG 1478 (Tocris, Bristol, United Kingdom), a reversible inhibitor used to prevent EGFR signaling prior to initiation of the experiment.

### 3.2 EGF immobilization

EGF was covalently immobilized in 24‐well tissue culture polystyrene plates (20 ng/well) via the heterobifunctional crosslinker Sulfo‐SANPAH.^8^, ^9^, ^35^ Briefly, 2.5 mM EGF in HEPES buffered saline solution (HBSS: 115 mM NaCl, 1.2 mM CaCl_2_ (Sigma), 1.2 mM MgCl_2_ (Sigma), 2.5 mM K_2_HPO_4_, 20 mM HEPES, pH adjusted to 7.6) was reacted with Sulfo‐SANPAH (SS) in a 1:50 M ratio for at least 3 hr covered in foil. The resulting photoactive SS‐EGF solution was then pipetted into each well (250 μL/well) and dried at 40°C for 6 hr. After drying, SS‐EGF was immobilized to the plate via exposure to 365 nm UV light for 120 s using an OmniCure® S2000 (EXFO, Inc., Chelmsford, MA), and unreacted EGF was removed by four rinses with diH_2_O on an orbital shaker. For control and soluble EGF conditions, plates were treated with HEPES buffered saline solution without the Sulfo‐SANPAH‐EGF mixture, dried at 40°C for 6 hr, and rinsed with diH_2_O on an orbital shaker.

### 3.3 Experimental conditions

HaCaTs were seeded at a sparse density in 24‐well plates prepared as described above with or without immobilized EGF at a final density of 50,000 cells/cm^2^. After 12 hr of cell attachment, cells were washed with phosphate buffered saline (PBS) and serum‐free media containing any treatments was added. To attain the confluent monolayer sheet, cells were added to each well of a fence device (catalog no. 80209; Ibidi GmbH, Fitchburg, WI) at a final concentration of 295,000 cells/cm^2^. Following 12 hr of cell attachment, sterile forceps were used to remove the device, cells were washed with PBS, and serum‐free media containing any treatments was added. For soluble conditions, this media was supplemented with 20 ng EGF/well, which we have previously determined provides an equivalent dose of EGF per cell as on the immobilized EGF.^9^ For control and immobilized conditions, this media was supplemented with 0.1% bovine serum albumin as a vehicle control. To disrupt tight junctions, cells were treated for 4 hr with 10 μM ochratoxin A (Sigma‐Aldrich, St. Louis, MO) or 0.1% DMSO as vehicle control. To initiate EGFR clustering, cells were treated for 4 hr with 50 mM methyl‐β‐cyclodextrin (Sigma‐Aldrich) or DMEM as a vehicle control.

### 3.4 Immunofluorescent staining

HaCaT cells were fixed upon fence removal or 4 hr later, following treatment with EGF, ochratoxin A, and/or methyl‐β‐cyclodextrin. All samples were fixed by incubation with 4% paraformaldehyde at room temperature for 15 minutes. After fixation, cells were washed with PBS and blocked in 5% goat serum and 0.3% Triton X‐100 in PBS for 1 hr at room temperature. Primary and secondary antibodies were diluted in 1% goat serum with 0.3% Triton X‐100 in PBS. Samples were incubated with primary antibodies overnight at 4°C, washed with PBS three times, and incubated with secondary antibodies for 2 hr at room temperature. Primary antibodies were rabbit anti‐pPLCγ1(Tyr783) (catalog no. 44696G, 1:200), mouse anti‐ZO‐1 (catalog no. 33‐9100, 1:100), rabbit anti‐EGFR (catalog no. 4267, all from Cell Signaling Technology, Danvers, MA, 1:100), and mouse anti‐EGFR (catalog no. ab30, Abcam, Cambridge, MA, 1:500). Secondary antibodies used were goat anti‐rabbit AlexaFluor488 (1:500, with rabbit anti‐pPLCγ1), goat anti‐rabbit AlexaFluor647 (1:125, with rabbit anti‐pPLCγ1), goat anti‐mouse AlexaFluor647 (1:500, with mouse anti‐ZO‐1 or 1:125, with mouse anti‐EGFR), or goat anti‐mouse AlexaFluor555 (1:125, with mouse anti‐ZO‐1 or mouse anti‐EGFR). Phalloidin counterstaining was done by incubation for 60 minutes with AlexaFluor594 phalloidin. Nuclei were counter‐stained with ProLong Diamond Antifade with DAPI.

### 3.5 Microscopy and image analysis

Cells imaged by confocal or STED were cultured on coverslips rather than in 24‐well plates. Coverslips (catalog no. 1404‐15; Globe Scientific, Paramus, NJ) were acid washed in 1 M HCl overnight on an orbital shaker, followed by four washes in tap water and two washes in diH_2_O for 10 minutes each. Coverslips were soaked in ethanol for 1 hr to sterilize and placed in a six well plate. The coverslips were further treated with Sulfo‐SANPAH‐EGF as necessary, and cells were seeded and treated as described in the manuscript methods.

Images in Supporting Information Figure 1 were collected on a Leica DMi8 inverted microscope (Leica, Buffalo Grove, IL). Images in Figures 1, 2, and 4d were collected on a Zeiss Axio Observer.Z1 inverted microscope with an AxioCam 506 mono camera, Plan‐NEOFLUOR 10× 0.3‐NA phase objective, 40× 0.6‐NA air objective, and Zen2 software (Zeiss; Oberkochen, Germany). Images in Figures 3, 4, 5 and Supporting Information Figures 2–3 were collected on a Leica SP8 inverted microscope (Leica; Wetzlar, Germany) equipped with a super‐continuum white‐light laser for fluorescent excitation from 470 to 670 nm and a separate 405 nm diode laser for confocal and three lasers (592 nm/660 nm/775 nm) for performing three‐color super‐resolution STED. A Plan‐APO 100× 1.4‐NA oil objective was used, and images were collected using three PMTs and two high‐sensitivity HyD detectors, and Leica LAX software. For confocal imaging, images were taken every 0.2 μm from the substrate/membrane interface until signal was lost at the top of the cell body.

All image analysis was performed with ImageJ (NIH, Rockville, MD). For pPLCγ1 quantification in Figure 1a, the average fluorescent intensity of pPLCγ1 was determined for each cell along the leading edge in control, soluble EGF, or immobilized EGF conditions at 4 hr. A minimum of 35 cells was measured across three or more images. Background levels were determined from the cell‐free region of the wound. For cell area analysis in Figure 1, phalloidin images from each condition at 4 hr were used to outline the cell boundary and measure the cell area. In Figure 2, cell boundaries were determined by outlining the cell boundary in the ZO‐1 channel, the fluorescent intensity per unit area for the pPLCγ1 channel was determined in this area, and background corrected using the fluorescent intensity per unit area in the cell‐free background from the same well. Cells with a fluorescent mean gray value of 1,200 or above was considered to be pPLCγ1 positive and below 1,200 to be negative. To determine if tight junctions were disrupted, fluorescent intensity values for ZO‐1 at the cell membrane and across the cytoplasm were determined (Figure 2b). Since ZO‐1 localization changes upon tight junction disruption,^36^ the ratios of fluorescent intensity between the cell membrane and cytoplasm were calculated to determine if cells had intact tight junctions (ratio ≥ 1.5) or disrupted tight junction (ratio < 1.5). To quantify EGFR localization from the basal to the apical side of the cell in Figure 3, a single cell was isolated from an image stack by hand drawing the cell outline, and fluorescent values at each z‐sections were measured using the “Plot z‐axis profile” command. In Figures 4 and 5, and Supporting Information Figure 2, EGFR clusters were measured by subtracting the fluorescence of nonclustered EGFR from the original image using the confocal image and the “Image Calculator” command, receptor clusters were identified using a (10/255) threshold, and the “Analyze Particles” command was used to measure the cluster sizes.

### 3.6 Statistical analysis

Data were analyzed with GraphPad Prism 7.0 (La Jolla, CA) and are presented as the mean ± *SD*, and all experiments were performed at least twice to ensure reproducibility. Statistical significance was determined using ANOVA with post‐tests as noted in the figure legends. In all tests, *p* < .05 was considered statistically significant; calculated *p* values and adjusted *p* values for multiple comparisons can be found in Supporting Information Table 1.

## AUTHOR CONTRIBUTIONS

C.S.K., X.Y., and S.J. performed the experiments; all authors participated in data analysis and figure preparation. C.S.K., K.S.M., and P.K.K. wrote the manuscript, and all authors reviewed the manuscript.

## CONFLICT OF INTEREST

The authors declare no competing financial interests.

## Supporting information


**Supporting Information Figure 1** PLCγ1 activation at the wound edge in response to control, soluble EGF, or immobilized EGF. HaCaTs were seeded as confluent monolayers and treated with vehicle control, soluble EGF, or immobilized EGF for 4 hr after the fence was lifted. Green indicates pPLCγ1, and blue is nuclear stain; dashed line indicates wound edge. Scale bar = 20 μm.
**Supporting Information Figure 2** STED imaging of cells in the bulk. (a) Cells in the bulk from all three treatment conditions in the wound model were examined by STED for EGFR at 4 hr. Scale bar = 3 μm. (b) Quantification of EGFR cluster sizes for each condition. Data presented as individual clusters (the number of clusters is noted for each condition), with mean ± *SD* shown as lines. Clusters were not significantly different for threshold of 0.1 μm^2^ by one‐sample *t* test, *p* < .05.
**Supporting Information Figure 3** STED imaging of cells in response to EGF and methyl‐β‐cylcodextrin (MβCD). (A) Overview of image analysis. To separate the clusters from general EGFR staining, the confocal image intensity was subtracted from the STED image, providing distinct clusters for automated detection. (B) Additional STED images demonstrating the range of cluster sizes observed and the increase in clustering with MβCD. Scale bar = 10 μm.
**Supporting Information Table 1** *p* value and adjusted *p* value for statistical comparisons in figures.
