# Supplementary material for: Leader cell PLCγ1 activation during keratinocyte collective migration is induced by EGFR localization and clustering
Source: Bioeng Transl Med. 2019 Jun 26;4(3):e10138. doi: 10.1002/btm2.10138 (PMC6764804; doi:10.1002/btm2.10138)
Supplement: Supplementary file 1 — Supporting Information Figure 1 PLCγ1 activation at the wound edge in response to control, soluble EGF, or immobilized EGF. HaCaTs were seeded as confluent monolayers and treated with vehicle control, soluble EGF, or immobilized EGF for 4 hr after the fence was lifted. Green indicates pPLCγ1, and blue is nuclear stain; dashed line indicates wound edge. Scale bar = 20 μm. Supporting Information Figure 2 STED imaging of cells in the bulk. (a) Cells in the bulk from all three treatment conditions in the wound model were examined by STED for EGFR at 4 hr. Scale bar = 3 μm. (b) Quantification of EGFR cluster sizes for each condition. Data presented as individual clusters (the number of clusters is noted for each condition), with mean ± SD shown as lines. Clusters were not significantly different for threshold of 0.1 μm2 by one‐sample t test, p < .05. Supporting Information Figure 3 STED imaging of cells in response to EGF and methyl‐β‐cylcodextrin (MβCD). (A) Overview of image analysis. To separate the clusters from general EGFR staining, the confocal image intensity was subtracted from the STED image, providing distinct clusters for automated detection. (B) Additional STED images demonstrating the range of cluster sizes observed and the increase in clustering with MβCD. Scale bar = 10 μm. Supporting Information Table 1 p value and adjusted p value for statistical comparisons in figures. [file BTM2-4-na-s001.docx]

**
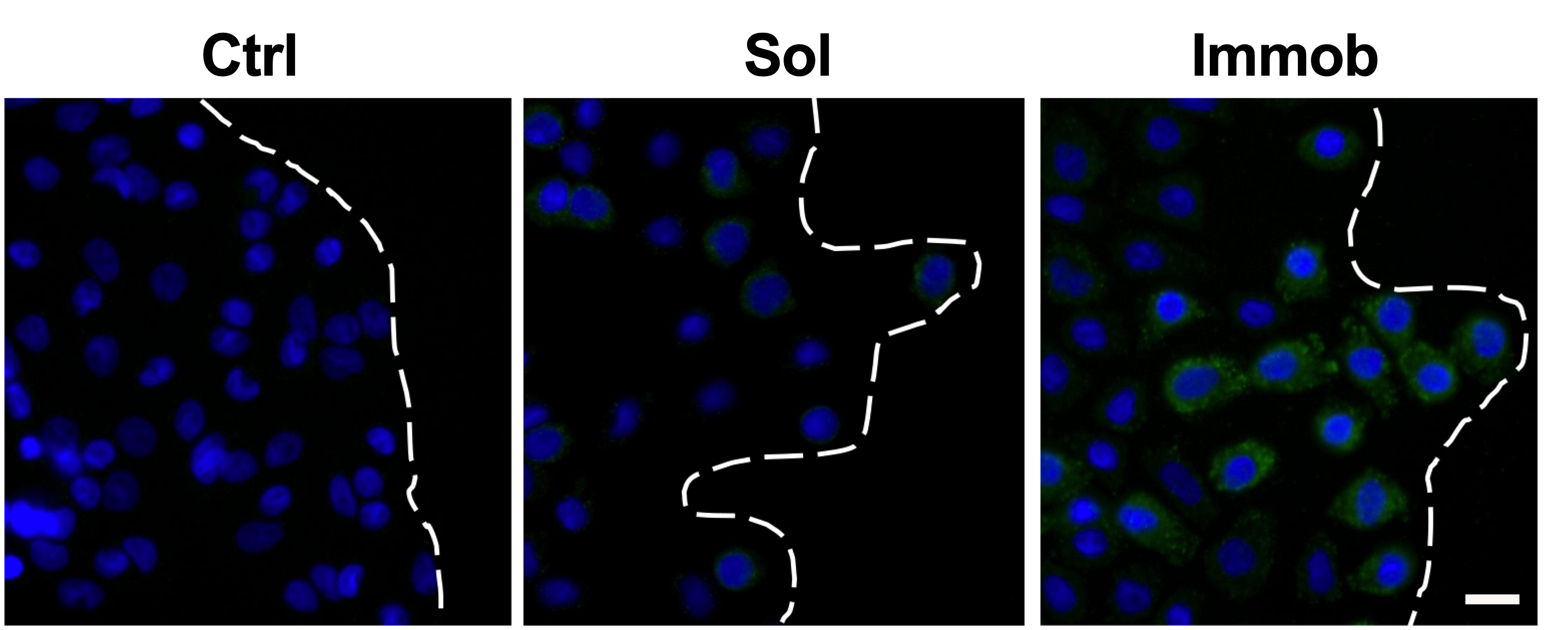
**

**Supplementary Figure 1. PLCγ1 activation at the wound edge in response to control, soluble EGF, or immobilized EGF.** HaCaTs were seeded as confluent monolayers and treated with vehicle control, soluble EGF, or immobilized EGF for four hours after the fence was lifted. Green indicates pPLCγ1, and blue is nuclear stain; dashed line indicates wound edge. Scale bar = 20 μm.


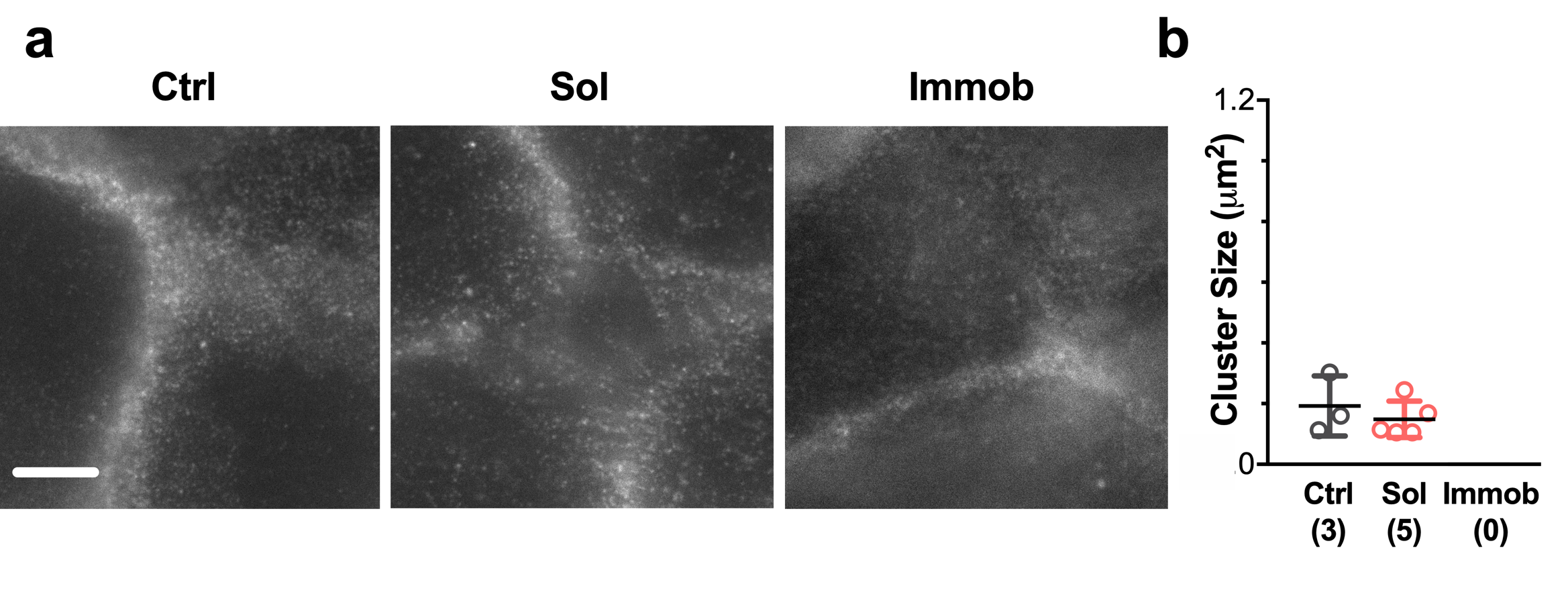


**Supplementary Figure 2. STED imaging of cells in the bulk.** **(a)** Cells in the bulk from all three treatment conditions in the wound model were examined by STED for EGFR at four hours. Scale bar = 3 μm. **(b)** Quantification of EGFR cluster sizes for each condition. Data presented as individual clusters (the number of clusters is noted for each condition), with mean +/- SD shown as lines. Clusters were not significantly different than threshold of 0.1 μm^2^ by one-sample t-test, p<0.05.

**
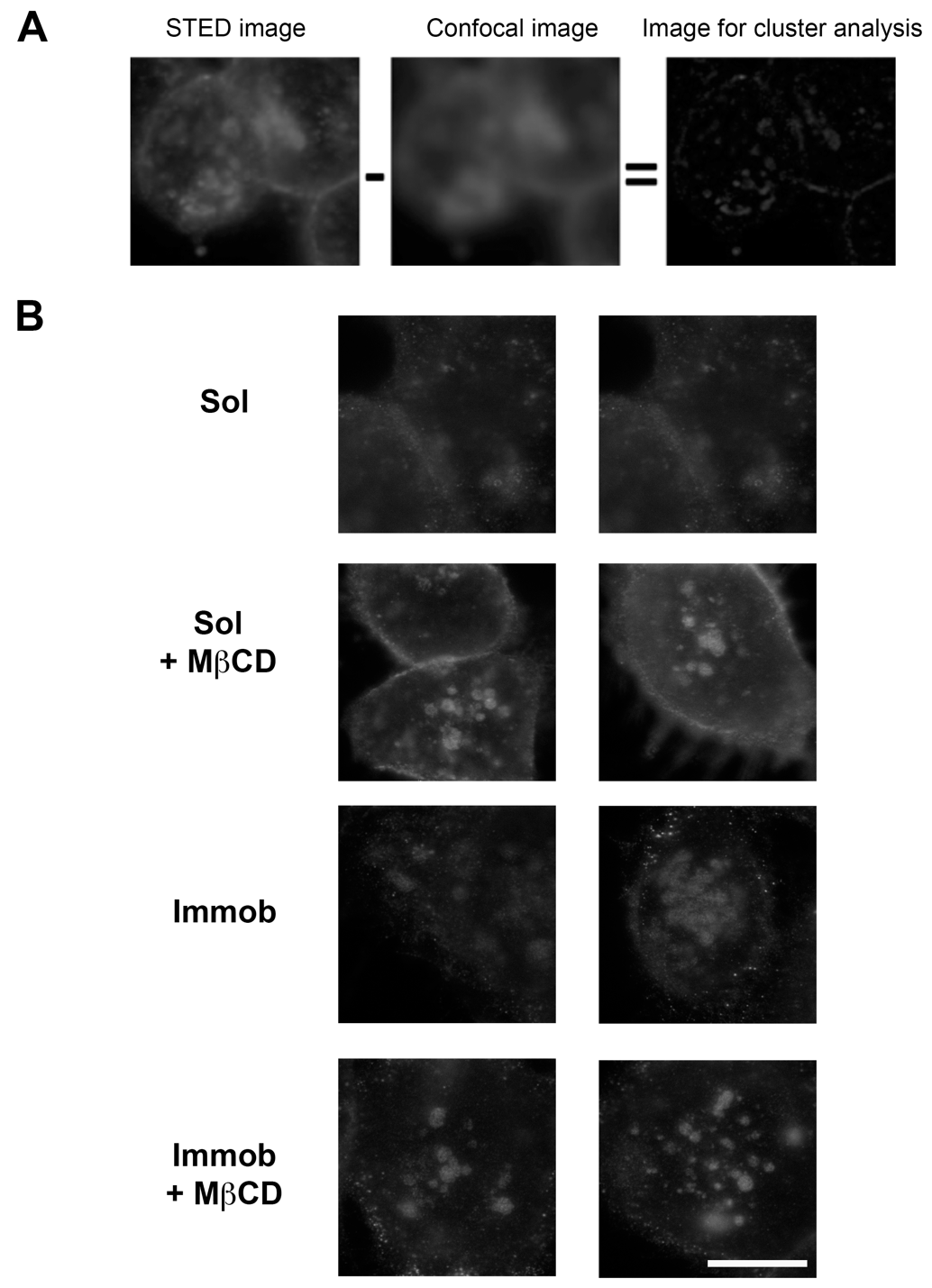
**

**Supplementary Figure 3. STED imaging of cells in response to EGF and methyl-β-cylcodextrin (MβCD).** **A)** Overview of image analysis. To separate the clusters from general EGFR staining, the confocal image intensity was subtracted from the STED image, providing distinct clusters for automated detection. **B)** Additional STED images demonstrating the range of cluster sizes observed and the increase in clustering with MβCD. Scale bar = 10 μm.

**Supplementary Table 1.** P-value and adjusted p-value for statistical comparisons in figures.

| **Figure** | **Comparison** | **p-value/adjusted p-value** |
| --- | --- | --- |
| Figure 1a | Ctrl. vs. Sol.  Ctrl. vs. Immob.  Sol. vs. Immob. | <0.0001  <0.0001  <0.0001 |
| Figure 1c | Ctrl. Bulk vs. Ctrl. Edge | <0.0001 |
|  | Ctrl. Bulk vs. Sol. Bulk | 0.92 |
|  | Ctrl. Bulk vs. Sol. Edge | <0.0001 |
|  | Ctrl. Bulk vs. Immob. Bulk | >0.99 |
|  | Ctrl. Bulk vs. Immob. Edge | <0.0001 |
|  | Ctrl. Edge vs. Sol. Bulk | <0.0001 |
|  | Ctrl. Edge vs. Sol. Edge | 0.097 |
|  | Ctrl. Edge vs. Immob. Bulk | <0.0001 |
|  | Ctrl. Edge vs. Immob. Edge | <0.0001 |
|  | Sol. Bulk vs. Sol. Edge | <0.0001 |
|  | Sol. Bulk vs. Immob. Bulk | 0.93 |
|  | Sol. Bulk vs. Immob. Edge | <0.0001 |
|  | Sol. Edge vs. Immob. Bulk | <0.0001 |
|  | Sol. Edge vs. Immob. Edge | <0.0001 |
|  | Immob. Bulk vs. Immob. Edge | <0.0001 |
| Figure 1e | PLCγ1 positive vs. PLCγ1 negative | 0.64 |
| Figure 3c | Ctrl. Bulk vs. Edge  Sol. Bulk vs. Edge  Immob. Bulk vs. Edge  Sol. Edge vs. Immob. Edge | <0.0001  <0.0001  <0.0001  0.31 |
| Figure 3e | 0 min Bulk vs. Edge  30 min Bulk vs. Edge  120 min Bulk vs. Edge | 0.18  0.22  0.010 |
| Figure 4 | Ctrl. vs. 0 (no test can be run as ctrl. is 0)  Sol. vs. 0  Immob. vs. 0  Sol. vs. Immob. | --  <0.0001  <0.0001  <0.0001 |
| Figure 5b | Ctrl. Veh. vs. MβCD | <0.0001 |
|  | Sol. Veh. vs. MβCD | <0.0001 |
|  | Immob. Veh. vs. MβCD | 0.011 |
| Figure 5c | Veh, Ctrl. vs. Veh, Sol. | 0.97 |
|  | Veh, Ctrl. vs. Veh, Immob. | 0.04 |
|  | Veh, Ctrl. vs. MβCD, Ctrl. | 0.92 |
|  | Veh, Ctrl. vs. MβCD, Sol. | <0.0001 |
|  | Veh, Ctrl. vs. MβCD, Immob. | <0.0001 |
|  | Veh, Sol. vs. Veh, Immob. | 0.21 |
|  | Veh, Sol. vs. MβCD, Ctrl. | 0.99 |
|  | Veh, Sol. vs. MβCD, Sol. | 0.0002 |
|  | Veh, Sol. vs. MβCD, Immob. | 0.0001 |
|  | Veh, Immob. vs. MβCD, Ctrl. | 0.32 |
|  | Veh, Immob. vs. MβCD, Sol. | 0.12 |
|  | Veh, Immob. vs. MβCD, Immob. | 0.1063 |
|  | MβCD, Ctrl. vs. MβCD, Sol. | 0.0003 |
|  | MβCD, Ctrl. vs. MβCD, Immob. | 0.0003 |
|  | MβCD, Sol.vs. MβCD, Immob. | >0.99 |
